# Asymmetry and structural system analysis of the proximal femur meta-epiphysis: osteoarticular anatomical pathology

**DOI:** 10.1186/1749-799X-3-11

**Published:** 2008-02-27

**Authors:** Ali A Samaha, Alexander V Ivanov, John J Haddad, Alexander I Kolesnik, Safaa Baydoun, Maher R Arabi, Irena N Yashina, Rana A Samaha, Dimetry A Ivanov

**Affiliations:** 1 Department of Anatomy, Faculty of Public Health, Lebanese University, Zahle, Lebanon; 2 Cellular and Molecular Signaling Research Group, Departments of Biological and Biomedical Sciences, Faculty of Arts and Sciences, Lebanese International University, Beirut, Lebanon; 3 Department of Anatomy, Kursk State Medical University, Russia; 4 Faculty of Arts and Sciences, Lebanese International University, Bekaa, Lebanon; 5 Clinical Laboratory, Faculty of Public Health, Lebanese University, Zahle, Lebanon

## Abstract

**Background:**

The human femur is commonly considered as a subsystem of the locomotor apparatus with four conspicuous levels of organization. This phenomenon is the result of the evolution of the locomotor apparatus, which encompasses both constitutional and individual variability. The work therein reported, therefore, underlies the significance of observing anatomical system analysis of the proximal femur meta-epiphysis in normal conditions, according to the anatomic positioning with respect to the right or left side of the body, and the presence of system asymmetry in the meta-epiphysis structure, thus indicating structural and functional asymmetry.

**Methods:**

A total of 160 femur bones of both sexes were compiled and a morphological study of 15 linear and angulated parameters of proximal femur epiphysis was produced, thus defining the linear/angulated size of tubular bones. The parameters were divided into linear and angulated groups, while maintaining the motion of the hip joint and transmission of stress to the unwanted parts of the limb. Furthermore, the straight and vertical diameters of the femoral head and the length of the femoral neck were also studied. The angle between the neck and diaphysis, the neck antiversion and angle of rotation of the femoral neck were subsequently measured. Finally, the condylo-diaphyseal angle with respect to the axis of extremity was determined. To visualize the force of intersystem ties, we have used the method of correlation galaxy construction.

**Results:**

The absolute numeral values of each linear parameter were transformed to relative values. The values of superfluity coefficient for each parameter in the right and left femoral bone groups were estimated and Pearson's correlation coefficient has been calculated (> 0.60). Retrospectively, the observed results have confirmed the presence of functional asymmetry in the proximal femur meta-epiphysis. On the basis of compliance or insignificant difference in the confidence interval of the linear parameters, we have revealed, therefore, a discrepancy in values between the neck and the diaphysis angle and the angle of femoral neck rotation (range displacement of confident interval to a greater degree to the right).

**Conclusion:**

This study assessed the observations of a systemic anatomical study encompassing the proximal femur meta-epiphysis behavior in normal condition. This work has significance in medical practice as the theoretical basis is also required in knowing the decreased frequency and degree of severity of osteoarthritic pathologies in the dominant lower extremity.

## Background

The femur, or as is commonly known as the thighbone, is one of the most thoroughly anatomically studied human body bones [1]. There is consensus as to the femur's anatomical peculiarities, age, gender and locomotion physiology [2]. Nevertheless, there is yet mounting controversy regarding the values of the linear and angular parameters of the proximal meta-epiphysis and their correlations.

The degree of the diaphysio-femoral neck angle according to Wagner and colleagues [3] varies from 125°3' to 132°3'. On the other hand, it was reported that the value may fluctuate from 109° to 153° [4], with no gender or racial predilection [5,6].

The antiversion angle range is approximately 74° (this value is conspicuously variable – it can vary from -12 to +74) [1]. Anatomically, it is well known that each skeletal bone is under certain influence of static and dynamic stress. This, in turn, defines the external shape and internal morphology of the femur's bone structure [7-11]. Nevertheless, the peculiarities of the femur and its epiphysis with regards to bilateral asymmetry (right or left side of the body) are not well understood [1,6].

We have previously reported the systematic organization of the femur [1], with subdivided groups into four levels of organization and anatomical values correlating with that of the human body joints. As the anatomy of the human body is characterized by the functional predominance of the right upper and left lower limbs [1,12-14], particular actuality was acquired in studying the value of parameters at different levels involved with forming the functional asymmetry of the femur bone [6].

The purpose of this work was to assess the observations of a systemic anatomical study encompassing the proximal femur meta-epiphysis behavior in normal condition. Our study has a spontaneous significance in medical practice as the theoretical basis is also required in unraveling the decreased frequency and degree of severity of osteoarthritic pathologies in the dominant lower extremity, in accordance with recurrent experimental observations [15-20].

## Materials and methods

### Sample collection and compilation

A total of one hundred and sixty (160) femur bones of both genders were compiled from a collection of human anatomy museums at the departments of several institutions, as previously indicated [1], without any indications of pathologic signs or symptoms or otherwise.

Furthermore, a morphological study of fifteen (15) linear and angulated parameters of proximal femur epiphysis was produced with the help of special arrangements [1], which allowed us to define the linear and angulated size of the tubular bones.

### Sample anatomical analysis

Depending on the degree of participation in function, all the investigated parameters of the proximal femur metaepiphysis were divided into linear and angulated groups, while maintaining the motion of the hip joint and transmission of stress to the unwanted parts of the limb.

Among the linear values that support the hip joint motion, we studied the straight and vertical diameters of the femoral head and the length of the femoral neck anteriorly, posteriorly, superiorly and inferiorly.

For the angulated values, we measured the angle between the neck and the diaphysis, the neck antiversion (rotation of the femoral neck in sagital plane), and angle of rotation of the femoral neck (in frontal plane). For the unwanted parts where the transmission of body weight occurs, we contributed the linear values as transverse size of the proximal epiphysis, and the vertical and straight neck diameters, intertrochanteric space, as straight and transverse diameter of diaphysis. Moreover, for the angulated values, we related the condylo-diaphyseal angle or angle of deviation of the femur with respect to the axis of extremity.

It is also noted that different ratios between various correlations with the value of ≥ 0.8 and < 0.7 at both groups (left and right bones) essentially indicate that the group of left bones is more specialized and thus functionally less universal.

### Statistical analysis

Results were assessed using the analysis software of Microsoft Excel XP and the method of correlation between systems and structures. In each group, the value of Pearson's correlation coefficient has been calculated among the studied parameters.

**Figure 1:**
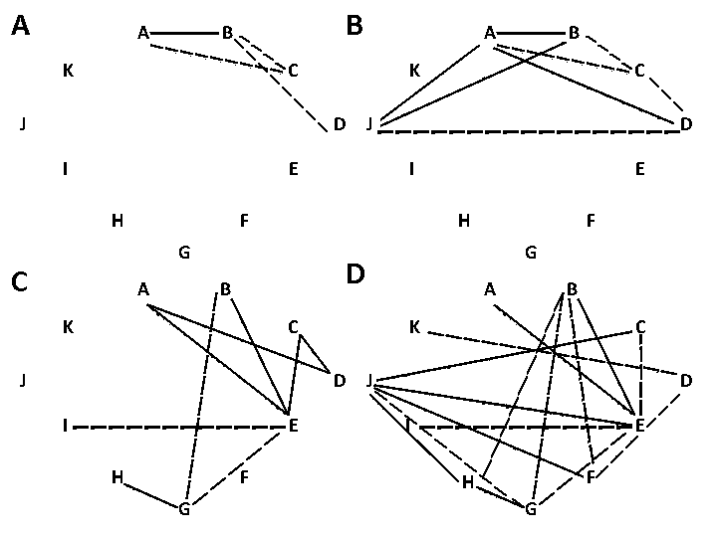
Correlation galaxies revealed during the structure analysis of the femur proximal meta-epiphysis (A, to the right; B, to the left; C, to the right; D, to the left). In figures 1A and 1B, ties with Pearson's correlation coefficient in the range of 0.8–0.89 are marked with dotted line; 0.9 and higher are marked with a continuous line. In figures 1C and 1D, ties with Pearson's correlation coefficient in the range of 0.6–0.69 are marked with dotted line; 0.7–0.79 are marked with a continuous line. Symbols: A – direct head diameter; B – vertical head diameter; C – direct neck diameter; D – vertical neck diameter; E – intertrochanteric size; F – front neck length; G – back neck length; H – upper neck length; I – lower neck length; J – proximal epiphysis transverse size; K – direct diaphysis diameter.

For the following analysis, correlation links have been taken into consideration with the correlation coefficient more than 0.6, as shown in Figure 1.

It's worth noting that all values were normalized (the procedure of dividing of the mean of each linear parameter by the mean of the transverse diameter of the femoral diaphysis). Therefore, the deviation of the measurement becomes irrelevant.

Furthermore, the value of the transverse diameter of the femoral diaphysis was used because this segment of the bone is specified for support (mono-functional).

To visualize the force of intersystem ties, we have used the method of correlation galaxy construction [1] (see Figure 1).

In accordance, each measurement (using our device and caliper) was produced four (4) times by one researcher and then the average values on each investigated linear or angular parameter were used for the following analysis procedures. As is well known, the repeatability of the measurement can be described and characterized directly or indirectly by several parameters, such as the standard deviation and dispersion. In our case, the repeatability of the measurement was dependent on two parameters: i) accuracy of the experimenter and ii) 'device mistake.'

Thus, one researcher and one device plus the following normalization process using the value of the transverse size of the femoral shaft (measures by a given experimenter and one caliper with the same accuracy and 'device mistake') indicate specific repeatability of a certain measurement. For example, the following relation indicates a specific degree of accuracy:

X (true value of any linear parameter) + x (current mistake of measurement)/D (true value of the transverse size of the femoral shaft) + d (current mistake of measurement) = A (normalized value of the measures linear parameter)

A = X + x/D + d

## Results

The absolute numerical values of each linear parameter were transformed to relative values (i.e., for each bone, the transverse diameter of diaphysis was considered a unit of measure), as shown in Table 1 (see *Statistical analysis *above). These parameters represent the absolute values of the intervals relating to the right and left femoral proximal meta-epiphysis bones, indicating proximity and specificity of the angular rotations (significance is realized at p ≤ 0.05).

**Table 1:** Parameters values of femoral proximal meta-epiphysis.*

Proximal meta-epiphysis parameters	Samples (n = 160)	Right femoral bones (n = 83)	Left femoral bones (n = 77)
Direct head diameter	1.63–1.69	1.63–1.71	1.60–1.70
Vertical head diameter	1.60–1.66	1.60–1.68	1.58–1.67
Transverse size	3.23–4.12	3.05–4.76	3.33–3.52
Front neck length	0.93–0.98	0.94–1.01	0.90–0.98
Back neck length	1.27–1.34	1.25–1.35	1.25–1.35
Lower neck length	1.51–1.60	1.51–1.64	1.46–1.59
Upper neck length	1.00–1.06	1.00–1.09	0.97–1.06
Diaphyseal neck angle	125.73–127.68	126.45–129.26	124.16–126.8
Anteversion neck angle	14.12–17.50	12.87–18.05	14.02–18.35
Rotation neck angle	20.66–22.65	21.29–24.11	19.16–21.91
Direct neck diameter	0.94–0.98	0.94–1.00	0.92–0.98
Vertical neck diameter	1.20–1.25	1.20–1.27	1.17–1.25
Intertrochanteric size	2.01–2.10	1.98–2.11	2.01–2.14
Direct diaphysis diameter	1.20–1.37	1.26–1.56	1.10–1.20
Condylo-diaphysial angle	8.96–9.60	9.02–9.88	8.62–9.59

* The value of the documented interval is given with Alpha being less than or equal to 0.05.

Furthermore, we have estimated the values of superfluity coefficient for each parameter in the right and left femoral bone groups, separately (the value of system information capacity). The results of the informational analysis are given in Table 2. The superfluity coefficient values of the researched hip arthrosis proximal meta-epiphysis parameters also indicate proximity and specificity.

**Table 2:** Superfluity coefficient values of the researched hip arthrosis proximal meta-epiphysis parameters.

Proximal meta epiphysis parameters	Right femoral bones (n = 83)	Left femoral bones (n = 77)
Direct head diameter	12.81	7.33
Vertical head diameter	9.7	10.04
Transverse size	12.95	6.27
Front neck length	24.54	7.21
Back neck length	15.89	15.69
Lower neck length	28.08	18.99
Upper neck length	27.37	17.26
Diaphyseal neck angle	8.59	19.97
Anteversion neck angle	20.15	23.24
Rotation neck angle	9.42	30.99
Direct neck diameter	8.78	8.28
Vertical neck diameter	7.56	13.20
Intertrochanteric size	9.57	9.85
Direct diaphysis diameter	30.00	9.93
Condylo-diaphysial angle	4.79	30.99

Furthermore, correlation analysis was undertaken. The values of Pearson's correlation coefficient among parameters of femoral bones proximal epiphysis are shown in Table 3. These correlation values among the aforementioned parameters of the femoral bones proximal epiphysis represent a correlating pattern characteristic of the right and left bones, diametrically and longitudinally.

**Table 3:** Values of correlation coefficient among parameters of femoral bones proximal epiphysis.*

		Pearson's Correlation Coefficient	
		
Correlating characteristics		Right Bones	Left Bones
Direct head diameter	Vertical head diameter	**0.94**	**0.98**
Direct head diameter	Direct neck diameter	**0.80**	**0.84**
Direct head diameter	Vertical neck diameter	0.76	**0.91**
Direct head diameter	Intertrochanteric size	0.77	0.74
Vertical head diameter	Direct neck diameter	**0.86**	**0.84**
Vertical head diameter	Vertical neck diameter	**0.80**	**0.90**
Vertical head diameter	Front neck length		0.61
Vertical head diameter	Upper neck length		0.61
Vertical head diameter	Back neck length	0.64	0.65
Vertical head diameter	Intertrochanteric size	0.79	0.76
Direct neck diameter	Vertical neck diameter	0.72	**0.81**
Direct neck diameter	Intertrochanteric size	0.71	0.68
Vertical neck diameter	Intertrochanteric size		0.67
Front neck length	Intertrochanteric size		0.60
Upper neck length	Back neck length	0.78	0.74
Lower neck length	Back neck length	0.67	
Back neck length	Intertrochanteric size	0.66	
Lower neck length	Intertrochanteric size	0.66	0.64
Transverse size	Direct head diameter		**0.92**
Transverse size	Vertical head diameter		**0.94**
Transverse size	Direct neck diameter		0.76
Transverse size	Vertical neck diameter		**0.88**
Transverse size	Front neck length		0.73
Transverse size	Upper neck length		0.72
Transverse size	Back neck length		0.68
Transverse size	Intertrochanteric size		0.77
Direct diaphysis diameter	Vertical neck diameter		0.66

* Cells with the bonding force of more than 0.8 are shown in bold; cells with the correlation coefficient less than 0.6 are shown in blank.

In each of the abovementioned analysis approaches, all absolute values were transformed to the relative type. This procedure, therefore, normalizes all values accordingly.

## Discussion

Retrospective review of the observed results confirms the presence of functional asymmetry in the proximal femur meta-epiphysis [1]. On the basis of compliance or insignificant difference in the confidence interval of the linear parameters, we have revealed a discrepancy in values between the neck and the diaphysis angle and the angle of femoral neck rotation (range displacement of confident interval to a greater degree to the right).

This fact can be explained by the obvious muscular imbalance and predominance of the right extremity in providing support function [18-24]. In the analysis of correlation dependence, we have not revealed any significant ties among angular and linear parameters. In our opinion, it indicates that their influence on the morphological and functional characteristics of the proximal femur meta-epiphysis is, in general, minimal and their absolute values characterize individual variability in the borders of the backbone (noted above) characteristics at the previous level [5-9].

Furthermore, we have revealed analytical correlation dependence (bonding force is more than 0.8) between the diameters of the femoral head and neck in both left and right bones groups (parameters are marked as A, B, C and D; Figure 1), which shows active participation of these structures in realizing the support function of the hip joint [1-5]. Besides, the given structures can be considered as backbones (system-organizing) [25-27]. The presence of correlation between the transverse size of the proximal epiphysis (J) and the diameter of the femoral head may indicate the predominance of the left extremity in providing movements in the hip joint and also the maintenance of the vertical position of body while walking [12-15].

Of particular significance, the results of the aforementioned informational analysis show that the femur proximal meta-epiphysis is asymmetric. Moreover, left proximal epiphysis has a greater margin of safety according to a number of parameters transmitting load to underlying leg part (vertical head and neck diameters, intertrochanteric space) and providing direct walking of a person (diaphyseal neck angle, neck anteversion and rotation angles) [2-7,16,27].

In addition to that, the results of the informational analysis and correlation ties of moderate intensity (Pearson's correlation coefficient 0.6–0.79) in both groups between the intertrochanteric space and the parameters of the femoral head confirm the hypothesis that the proximal parts of the femur act at a level that transmits load to the knee joint [28-31].

The centre of the femoral head is the place of strength application that leads to the development of significant flexion; its value can be defined as the distance between linear action of strength and axis of the center of bone gravity [25-31]. Moreover, there are three types of tension in bones: flexion, compression and rotation [32]. An additional bone compression occurs on the side of strength action, whereas a stress sprain develops on the opposite side.

Transmission of the axial load to the hip joint region occurs in different positions – it can be adducted and abducted in many directions (anterior, posterior, etc.) [32]. Furthermore, stress on the diaphysis is transmitted through the head by means of neck. Biomechanical stress axis may also form an angle with the anatomical axis [1].

In case of maximal femur adduction there will be more eccentricity, where in the subtrochanteric area more flexion is seen [27-32]. On the left, correlation ties between the intertrochanteric space and the transverse size of the proximal epiphysis (marked as E and J) confirm this hypothesis and show a greater degree of fulfillment of the support and moving function of the left leg.

On the basis of the aforementioned analysis, we can formulate the conclusion that there is a system asymmetry of the proximal femur in normal condition with the predominance of the left proximal epiphysis in providing moving and support function. The right proximal femur meta-epiphysis is less adjusted to movement and severe strain. This indicates the prevalence of degree and frequency of the right hip joint impairment [33-36].

In accordance with the aforementioned, it can be concluded that the less the number of correlating values amongst 'right-side' parameter means, the more the right femur is functionally 'universal,' less 'structural'. This thereby exhibits the realization of more functions as compared with the left bone [1].

## Competing interests

The author(s) declare that they have no competing interests.

## Authors' contributions

All authors have squarely and equally contributed to developing the experimental, theoretical and statistical aspects of this article.
